# The Relationship Between Insufficient Vision and Technology Access and Use Among Hospitalized Adults at an Urban Academic Hospital: Observational Study

**DOI:** 10.2196/40103

**Published:** 2023-05-24

**Authors:** Juhi C Gupta, Vineet M Arora, Hanna Vollbrecht, Nicole Kappel, David O Meltzer, Valerie G Press

**Affiliations:** 1 University of Chicago Pritzker School of Medicine Chicago, IL United States; 2 Section of General Internal Medicine Department of Medicine University of Chicago Chicago, IL United States; 3 Department of Medicine Brigham and Women’s Hospital Boston, MA United States; 4 Section of Hospital Medicine Department of Medicine University of Chicago Chicago, IL United States

**Keywords:** vision, health technology, chronic disease, ownership, internet, eHealth, digital health, eye, optometry, myopia, ophthalmology, myopic, digital device, observational study, use, visual impairment, visually impaired

## Abstract

**Background:**

The role of sufficient vision in self-management is salient with respect to the growing prevalence of eHealth-based interventions for chronic diseases. However, the relationship between insufficient vision and self-management has been understudied.

**Objective:**

We aimed to assess differences in access to and use of technology among adults with and without insufficient vision at an academic urban hospital.

**Methods:**

This is an observational study of hospitalized adult general medicine patients that is part of a larger quality improvement study called the hospitalist study. The hospitalist study provided demographic and health literacy data (Brief Health Literacy Screen). Our substudy included several measures. Validated surveys assessed technology access and use, and included benchmarked questions from the National Pew Survey to determine access to, willingness to use, and self-described ability to use technology at home, particularly for self-management, and eHealth-specific questions assessing future willingness to access eHealth post discharge. The eHealth Literacy Scale (eHEALS) was used to assess eHealth literacy. Visual acuity was assessed using the Snellen pocket eye chart with low vision defined as visual acuity ≤20/50 in at least one eye. Descriptive statistics, bivariate chi-square analyses, and multivariate logistic regressions (adjusted for age, race, gender, education level, and eHealth literacy) were performed using Stata.

**Results:**

A total of 59 participants completed our substudy. The mean age was 54 (SD 16.4) years. Demographic data from the hospitalist study was missing for several participants. Among those who responded, most identified as Black (n=34, 79%) and female (n=26, 57%), and most reported at least some college education (n=30, 67%). Most participants owned technology devices (n=57, 97%) and had previously used the internet (n=52, 86%), with no significant differences between those with insufficient and sufficient vision (n=34 vs n=25). Though there was a 2x effect size for laptop ownership, with those with sufficient vision more likely to own a laptop, those with insufficient vision versus sufficient vision were less likely to report an ability to perform online tasks without assistance, including using a search engine (n=22, 65% vs n=23, 92%; *P*=.02), opening an attachment (n=17, 50% vs n=22, 88%; *P*=.002), and using an online video (n=20, 59% vs n=22, 88%; *P*=.01). In multivariate analysis, the ability to independently open an online attachment did not remain statistically significant (*P*=.01).

**Conclusions:**

Technology device ownership and internet use rates are high in this population, yet participants with insufficient vision (vs sufficient vision) reported a reduced ability to independently perform online tasks. To ensure the effective use of eHealth technologies by at-risk populations, the relationship between vision and technology use needs to be further studied.

## Introduction

Sufficient vision, a lack of uncorrectable loss of vision interfering with daily activities, is important in chronic disease self-management including medication adherence [1,2]. Forty percent of older American adults have mild visual impairment [3]. Vision decline is commonly associated with aging and eye diseases (eg, cataracts, glaucoma, age-related macular degeneration, diabetic retinopathy) [4]. Potential challenges related to vision loss include difficulties with functional activities (eg, mobility, personal care), social interaction, emotional distress, delirium, difficulty reading health information (eg, consent forms, discharge instructions), and decreased quality of life in the inpatient setting [5]. Insufficient vision is associated with low health literacy (HL) and may hinder the self-management of chronic diseases [6]. For example, persons with insufficient vision may take longer to read and understand health information [7].

In recent years, the prevalence of chronic disease self-management eHealth interventions has increased [8,9]. Many eHealth interventions, including mobile health (eg, SMS text message or smartphone app based), interactive video modules (eg, Virtual Teach-To-Goal programs), and wearable technologies (eg, smartwatches), were piloted and showed improvement in at-home self-management [6,10-15]. At-home eHealth interventions can be convenient, accessible, and low-cost for many people living in the United States [13]. However, adequate eHealth literacy (eHL), a distinct type of literacy [9,16], may be necessary to effectively use such interventions. Importantly, studies rarely assess the feasibility of eHealth interventions in persons with insufficient vision. The COVID-19 pandemic has raised concerns regarding health care access among individuals with low vision. Vaccine distribution across major cities relies heavily on access to online portals [17]. Furthermore, at-home COVID-19 testing is largely inaccessible to the blind community in the United States given difficulties using technology [18]. Hence, those with insufficient vision may have underrecognized barriers to accessing and using eHealth. Therefore, we studied access to and use of technology among adult inpatients with and without insufficient vision at an academic urban hospital.

## Methods

### Overview

This cross-sectional observational study was a substudy of an ongoing quality-of-care study of adult inpatients admitted to general medicine (hospitalist study) [19]. The hospitalist study provided demographic and HL data (Brief Health Literacy Screen) [20]. Our substudy included several measures. Validated surveys assessed technology access and use, and included benchmarked questions from the National Pew Survey to determine access to, willingness to use, and self-described ability to use technology at home, particularly for self-management [21], and eHealth-specific questions assessing future willingness to access eHealth post discharge. The eHealth Literacy Scale (eHEALS) was used to assess eHL [22].

Visual acuity was measured using a Snellen pocket eye chart, with insufficient vision defined as visual acuity ≤20/50 in at least one eye, chosen based on its association with decreased functional status in senior patients [23]. Room conditions were optimized prior to vision screening, including minimizing distractions like television and ensuring optimal room brightness. The participants wore personal corrective lenses during the vision screening, if available and applicable. The Snellen pocket eye chart was held by the researcher 14 inches away from the participant’s forehead at eye level. Each eye was covered by the participant using an occluder and placed underneath corrective lenses, if applicable. Participants were asked to read the row of words from left-to-right when measuring visual acuity in the right eye and right-to-left when measuring visual acuity in the left eye starting with the largest font first. Data were collected and managed using REDCap version 11.1.7 (Vanderbilt University) [12].

Descriptive statistics, bivariate chi-square analyses, and multivariate logistic regressions (adjusted for age, race, gender, education level, and eHL) were performed using Stata version 15 (StataCorp). All demographic data were tested against a Bonferroni-adjusted alpha level of .006 (.05/8). Device ownership analyses were tested against a Bonferroni-adjusted alpha level of .008 (.05/6). All technology use analyses were tested against a Bonferroni-adjusted alpha level of .008 (.05/6).

### Ethics Approval

The University of Chicago Biological Sciences Division Institutional Review Board (hospitalist survey IRB16-1131 and technology survey IRB16-0763) approved this study. Trained researchers screened and approached eligible patients from June 2019 to January 2020. Patients were eligible if they met the following criteria: enrolled in the hospitalist study, age ≥18 years, English speaking, no need for proxy, on general medicine services, and provided informed consent. Study data have been deidentified.

## Results

**Figure 1:**
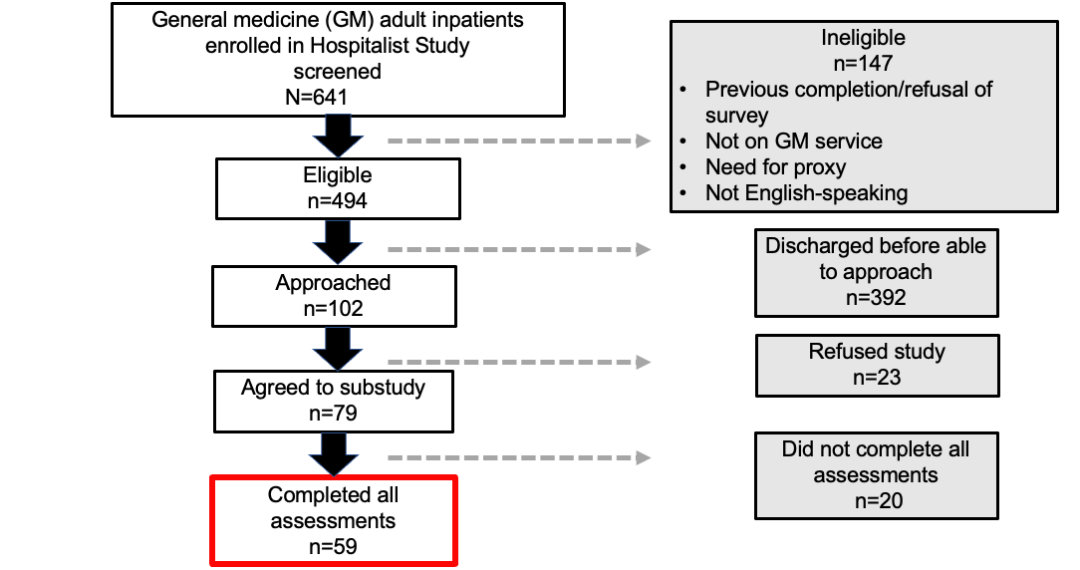
CONSORT (Consolidated Standards of Reporting Trials) diagram. CONSORT diagram for participant enrollment in our study. The gray boxes represent participants who were not included in the final analysis. We screened 641 patients who were enrolled in the hospitalist study and had a total of 59 participants complete all surveys.

A total of 59 participants were enrolled in our substudy (Figure 1). We were missing demographic data from the main hospitalist study for several participants. Of the 43 with race data, most identified as Black (n=34, 79%); of the 46 participants with gender data, over half (n=26, 57%) identified as female; and of 45 with education data, most reported some college education (n=30, 67%). In the total study population, the mean age was 53 (SD 16.4, range 19-85) years. About one-quarter (n=15, 26%) had low eHL, and 3% (n=2) had low HL (Table 1).

Over half (n=34, 58%) had insufficient vision (Table 1). Less than half (n=27, 46%) wore corrective lenses during vision screening; others reported not regularly wearing corrective lenses (n=11, 26%) or not having access to or choosing not to wear corrective lenses during screening (n=8, 14%). Less than one-quarter (n=14, 24%) reported an eye disease diagnosis (eg, cataracts).

Most (n=57, 97%) owned at least one technology device. There were no significant differences in device ownership between participants with sufficient and insufficient vision (n=25, 100% vs n=32, 94%; *P*=.22), including with specific devices: desktop (n=9, 36% vs n=13, 38%; *P=*.86), laptop (n=16, 64% vs n=13, 38%; *P=*.05), tablet (n=15, 60% vs n=16, 47%; *P=*.33), cell phone (n=5, 20% vs n=3, 9%; *P=*.22), and smartphone (n=21, 84% vs n=28, 82%; *P=*.87; Table 2). Though, there was nearly a 2x effect size between groups for laptop ownership.

Most had previously used the internet (n=52, 88% overall; n=28, 83% insufficient vs n=24, 96% sufficient vision; *P*=.10). Those with insufficient vision (vs sufficient vision) reported a significantly lower ability to open an attachment (n=17, 50% vs n=22, 88%; *P*=.002). In multivariate analysis, the decreased ability to independently open an online attachment for persons with insufficient vision was not significant when adjusting for age, gender, race, education, eHL, and any diagnosis of eye disease (*P*=.01; Table 3).

**Table 1:** Study population demographic data^a^.

Characteristic		All (N=59)	Insufficient vision (n=34)	Sufficient vision (n=25)	*P* value	
Age (years), mean (SD)		53 (16.4)	58 (13.0)	46 (18.0)	.004^b^	
Age ≥65 years, n (%)		14 (24)	10 (30)	4 (16)	.23	
Female gender^c^, n (%)		26 (57)	14 (58)	12 (55)	.80	
**Race^d^, n (%)**						.78
	Black	34 (79)	17 (77)	17 (81)		
	White	8 (19)	5 (28)	3 (14)		
**Education^e^, n (%)**						>.99
	High school or less	15 (3)	8 (33)	7 (33)		
	At least some college	30 (67)	16 (67)	14 (67)		
Low health literacy^e^, n (%)		1 (2)	14 (41)	0 (0)	.34	
Low eHealth literacy, n (%)		15 (26)	11 (32)	4 (16)	.22	
**Eye disease diagnosis, n (%)**		14 (24)	10 (29)	4 (16)	.23	
	Cataracts	10 (17)	7 (21)	3 (12)		
	Retinopathy	0 (0)	0 (0)	0 (0)		
	Other eye disease	7 (12)	5 (15)	2 (8)		

^a^Bonferroni correction α level .006 (.05/8).

^b^Significant at *P*<.006.

^c^Data obtained from 46 total participants; other data missing.

^d^Data obtained from 43 total participants; other data missing.

^e^Data obtained from 45 total participants; other data missing.

**Table 2:** Technology device ownership by visual acuity^a^.

Device ownership	All (N=59), n (%)	Insufficient vision (n=34), n (%)	Sufficient vision (n=25), n (%)	*P* value
Any device	57 (97)	32 (94)	25 (100)	.22
Desktop	22 (37)	13 (38)	9 (36)	.86
Laptop	29 (49)	13 (38)	16 (64)	.05
Tablet	31 (53)	16 (47)	15 (60)	.33
Smartphone	49 (83)	28 (82)	21 (84)	.87
Cell phone (nonsmartphone)	8 (14)	3 (9)	5 (20)	.22

^a^Bonferroni correction alpha level .008 (.05/6). Rates of technology device ownership in the general population (all), the insufficient vision group, and the sufficient vision group. Rates of device ownership are similar across all groups, with no significant differences in ownership.

**Table 3:** Self-described ability to use the internet^a^.

Characteristic			All (N=59), n (%)	Insufficient vision (n=34), n (%)	Sufficient vision (n=25), n (%)	*P* value
Ever used internet			52 (88)	28 (83)	24 (96)	.11
**Internet capabilities**						
	Use search engine		45 (76)	22 (65)	23 (92)	.02
	Open an attachment		39 (66)	17 (50)	22 (88)	.002^b,c^
	Print web pages/online information		30 (51)	15 (44)	15 (60)	.23
	Upload images/files to a website		36 (61)	19 (56)	17 (68)	.35
	**Use a video**		42 (71)	20 (59)	22 (88)	.01
		Use an interactive video	35 (59)	17 (50)	18 (72)	.09

^a^Bonferroni correction α level .008 (.05/6). Participants in the insufficient vision group reported decreased self-described ability to open an attachment and use a video online as compared to participants with sufficient vision.

^b^Significant at *P*<.006.

^c^When adjusting for age, race, gender, education, eHealth literacy, and any diagnosis of eye disease, opening an attachment did not remain significant (*P*=.01).

## Discussion

In this small study, we found high levels of device ownership and internet use overall. Despite this, participants with insufficient vision reported reduced ability to perform online tasks compared to those with sufficient vision.

High device ownership and internet use rates in our population suggest that technology and the internet may be feasible for administering self-management interventions for many individuals depending on the setting and type of technology used. For example, a Virtual Teach-To-Goal program (completed on devices with the internet) taught inhaler skills to patients with asthma or chronic obstructive pulmonary disease and improved mastery and confidence skills post intervention during hospitalization at the same institution as our study [11]. Additionally, an SMS text message–based intervention (not requiring internet access) was a feasible and useful approach to improving diabetes self-management in an urban Black population [14]. As demonstrated in this study, tailoring of when, where, how, and on what devices is likely needed, particularly among individuals with insufficient vision.

Among some subpopulations, technology-based interventions may not be useful or accessible. In 2019, 25% of adults aged ≥65 years did not use the internet and half did not own a smartphone [17]. Older age and age-related diseases (eg, macular degeneration and glaucoma) are associated with a vision decline [24]. Older adults, therefore, are particularly at risk for the inability or ineffective use of eHealth interventions. In the general population, though there is high device ownership in general, digital redlining or high costs of data may limit device use for time-intensive or even brief interventions [17,25]. Additionally, low HL and eHL are important barriers to online use capabilities [26,27]. Insufficient vision is correlated with decreased HL, specifically increased time to read and understand health information, which may further hinder ability to use self-management interventions [7].

These findings highlight insufficient vision as an underrecognized barrier to eHealth use. In our study, participants with insufficient vision reported difficulty independently using key features of the internet. Many current eHealth interventions may not consider that insufficient vision can lead to difficulty interacting with essential information. For example, people with blindness reported challenges using at-home COVID-19 tests, including difficulties with accessing websites selling at-home tests and using tests due to using smartphones for audio instructions [18]. Future eHealth intervention designers could consider optimizing the interface to improve usability for those with insufficient vision. For example, larger text font sizes, supplementary audio narrations, and simplified funnels may decrease the necessity for sufficient vision to use eHealth. Another solution is regular vision screening, so education can be tailored to fit patients’ unique needs.

Although this study identified insufficient vision as an important barrier to using technology-based interventions for self-management, it is important to note the study’s limitations. First, the sample size was smaller than planned because the COVID-19 pandemic interrupted certain study protocols including vision testing. Moreover, we had a small percentage of our population with self-reported low HL, thus we could not assess the relationship between insufficient vision and low HL. Persons with low HL may be less interested in participating in research studies, thus the low prevalence of low HL in our population may be due to selection bias [28]. Next, given that this is a single-site study with a predominantly urban Black population, the findings may be limited to similar populations and, therefore, have less large-scale generalizability. However, given that this is an understudied population, these results are valuable in informing interventions for this group. The surveys are also based on self-report, resulting in potential recall bias. Finally, visual acuity data may be affected because not all patients who required corrective lenses wore them during screening.

In summary, our findings could inform the conceptualization, development, and implementation of self-management eHealth interventions for people with insufficient vision. To ensure at-risk populations can use disease management and health-enhancing technologies, future studies must be conducted to better understand the relationship between vision and eHealth use.

## Abbreviations

eHEALS: eHealth Literacy Scale
eHL: eHealth literacy
HL: health literacy

## Data Availability

Since the data used in this manuscript are highly granular and contain potentially sensitive patient information, public sharing of the data would breach the University of Chicago’s Institutional Review Board protocol requirements. Interested researchers may contact VGP via email at vpress@medicine.bsd.uchicago.edu for data requests.
